# miR-769-3p inhibits cellular proliferation of KSHV-infected SH-SY5Y cells through targeting mTOR

**DOI:** 10.7150/jca.93595

**Published:** 2024-04-23

**Authors:** Dongdong Cao, Zhaofu Wu, Rui Yang, Lixia Yao, Jinhong Huang, Yufei Ding, Aynisahan Ruzi, Zemin Pan, Yuanming Pan, Dongmei Li, Wenyi Gu, Jinli Zhang

**Affiliations:** 1 School of medicine, Shihezi University/Laboratory of Xinjiang Endemic and Ethnic Diseases, Ministry of Education,832002, Xinjiang, China.; 2 Australian Institute for Bioengineering and Nanotechnology (AIBN), University of Queensland (UQ), St Lucia, Brisbane QLD 4072, Australia.; 3 Department of Pathology, Yili Friendship Hospital, 835099, Xinjiang, China.; 4 Department of Pathology, Bazhou Hospital, 841000, Xinjiang, China.; 5 The Affiliated Hospital of Hubei Provincial Government/Hubei Rehabilitation Hospital, 430064, Hubei, China.; 6 Cancer Research Center, Beijing Chest Hospital, Capital Medical University, Beijing Tuberculosis and Thoracic Tumor Research Institute, No. 9 Beiguan Street, Tongzhou District, Beijing 101149, China.

**Keywords:** Kaposi's sarcoma-associated herpesvirus, miR-769-3p, mTOR, cell proliferation, cell migration

## Abstract

The infection by Kaposi's sarcoma-associated herpesvirus (KSHV) is one of the most common causes of death in AIDS patients. Our studies have found that KSHV can infect SH-SY5Y cells (named SK-RG) *in vivo* and mTOR was up-regulated, which results in remarkable enhancement of cell proliferation, migration. But the regulatory role of mTOR in KSHV infected neurons has not yet been fully elucidated. Here, we find that miR-769-3p is decreased in SK-RG cells, which can exert anti-KSHV effect through negatively regulating the expression of mTOR. The knockdown of mTOR or overexpress of miR-769-3p decreased the proliferation, migration ability and cell cycle related protein of SK-RG cells, and the expression of KSHV related genes. In contrast, activating mTOR function by 3BDO treatment weakened the cellular behaviors of miR-769-3p overexpressing cells. Meanwhile, overexpressed miR-769-3p and rapamycin showed a shared inhibition trend in the effects on cell proliferation and motility. Our data indicated that miR-769-3p can inhibit cell proliferation and migration by down regulating mTOR in KSHV infected SH-SY5Y cells, and can be a candidate molecule for anti-KSHV therapy.

## 1. Introduction

Kaposi's sarcoma-associated herpesvirus (KSHV) is also known as humanherpesvirus-8 (HHV-8). In 1994, the virus was first isolated from Kaposi's sarcoma (KS) of AIDS patients by Chang *et al.* 1. KSHV is a double-stranded DNA virus belonging to the gamma-herpesvirus, and has been proved to be associated with proliferative diseases such as Kaposi's sarcoma, Multicentre Casteman disease (MCD) and primary effusion lymphoma (PEL) 2-4. In recent years, some researches have reported that the herpesvirus family of KSHV has a neurotropic tendency 5, 6. AIDS patients are susceptible to KSHV infection and often have symptoms related to the neuropathy, such as memory loss, abnormal behavior, and mental decline 7-9.

To further explore the role of KSHV in the nervous system, we infected SH-SY5Y cells by KSHV *in vitro* and successfully established a cell line named SK-RG. KSHV in infected cells was found to undergo spontaneous lytic activation without induction 6, 10. However, the mechanism of action of KSHV on the host's central nervous system and related nerve cells is still unclear. We found that in the differentially expressed genes provided by the transcriptome sequencing, mTOR was up-regulated in SK-RG cells. mTOR also plays a key role in the development of nervous system, such as participating in the regulation of the cell proliferation and differentiation, and the formation of neural synapses 11, 12. Moreover, the dysregulated activity of mTOR is involved in many pathophysiological conditions, such as aging, Alzheimer's disease, diabetes, obesity, and cancer formation 13-15. However, the role of mTOR in KSHV-infected neurons is still unknown.

microRNAs are small endogenous non-coding RNAs that target the 3'-untranslated region (3'-UTR) of mRNAs and then regulate their expressions 16, 17, which play an important role in cellular development, proliferation, and differentiation, their imbalance therefore involves in the development of a variety of cancers 18-21. miRNAs usually act as oncogenes or tumor suppressors by regulating the expression levels of key proteins 22, 23. There are also some miRNAs that can regulate the expression of mTOR, which is a key molecule in PI3K/AKT pathway for cell survive 24-26. Thus, we used Target scan 3.0, RNAhybrid 2.2, and miRanda to make predictions and screened out a miRNA, miR-769-3p. So far, there are only few reports on the function of miR-769-3p, but it has been shown to inhibit the growth and metastasis of tumors 26-28, and it has the potential value in the neuro tumor. But whether miR-769-3p can target mTOR and has the positive or negative function in the KSHV infected cells is still unknown.

Here, we examined the ability of proliferation and migration of SK-RG cells after knocking down mTOR or overexpressing miR-769-3p. The targeted regulation of miR-769-3p on mTOR and its role in SK-RG cells were verified. We also used mTOR activator to carry out the rescue assay and used mTOR inhibitor to study the effects of miR-769-3p, the results indicate that mTOR may be a promising target for the treatment of neurological diseases caused by KSHV infection and miR-769-3p could be a candidate molecular for anti-KSHV therapy.

## 2. Materials and Methods

### 2.1 Cell culture

SH-SY5Y and 293T cell lines were purchased from Shanghai Cell Bank, Chinese Academy of Sciences. SH-SY5Y and 293T cells were cultured in complete medium prepared with pure DMEM (Gibco), 10% FBS (Hyclone), and 1% penicillin-streptomycin (Solarbio), SK-RG cells were cultured in completed medium containing 6 μg/mL puromycin (Sigma) at 37 °C, 5% CO_2_ incubator.

### 2.2 RNA extraction and real-time PCR

The total RNA of SH-SY5Y, SK-RG and transfected cells were extracted with the Trizol reagent (Invitrogen), and the RNA concentration was measured. cDNA was synthesised by reverse transcription kit (Thermo Fisher), and the target fragment amplified by real-time PCR according to the reaction conditions as described in previous study^29^. Primer sequences are shown in Table 1.

### 2.3 Cell transfection

Using Lipofectamine 2000 (Invitrogen) to transfected si-mTOR, miR-769-3p mimics and corresponding negative controls (Genepharma). The complete medium was replaced after transfection 5h, and the cells were collected for further analysis after 48h.

### 2.4 Cell proliferation assay

After transfection for 24h, we seeded SK-RG cells into a 96-well plate. We added 20 μl (5mg/ml) of MTT solution to the wells at different time point, and then continued the cell incubation for 4 h at 37 °C, 5% CO_2_ incubator. We then removed the supernatant and added 150μl of DMSO to mix about 15 minutes at room temperature, and following that detected the absorbance value at 490 nm.

### 2.5 Plate cloning assay

After culturing the transfected SK-RG cells for 24h, the cells were seeded into a 6-well plate (600 cells per well). The medium was changed every 3 days. Incubated for 14 days and removed the supernatant. 4% paraformaldehyde was used to fixed the cells for 30min, and then 0.1% crystal violet (Solarbio) was used to stain for 30 min. After the 6-well plate was air-dried, we calculated the number of cell clones by microscope.

### 2.6 Cell scratch assay

We cultivated the transfected SK-RG cells to a density of 90%, used a 1ml sterile pipette tip to streaked a horizontal line at the bottom of the six-well plate, washed twice with 1×PBS, and added 2ml of medium containing 3% serum. The scratches at the same position were photographed at 0 h, 36 h, and 48 h.

### 2.7 Cell cycle analysis

After culturing the transfected SK-RG cells for 48 h, we collected and slowly added it into ethanol (70%) and stored at -20°C for 2 days. The supernatant after centrifugation was discarded, 1×PBS buffer was added and mixed well and stood for 15min. We centrifuged the cells to discard the supernatant. We added 500ul DNA staining solution and mixed well. We incubated the cells in the dark for 30min, and used flow cytometer (BD Bio-sciences) to detect and analyze.

### 2.8 Transwell assay

After culturing the transfected SK-RG cells for 24 h, the cells were collected. 20,000 cells were seeded into the upper chamber of a transwell plate, and 600ul of DMEM containing 15% serum was added to the lower chamber. After culturing for 48 h, paraformaldehyde was used to fix the cell for 30 min, and 0.1% crystal violet was used to stain. Three areas were randomly selected to count the number of migrated cells.

### 2.9 Dual-luciferase reporter gene assay

We set up four groups: pmirGLO-mTOR-wt+NC mimics, pmirGLO-mTOR-wt+miR-769-3p mimics, pmirGLO-mTOR-mut+NC mimics and pmirGLO-mTOR-mut+miR-769-3p mimics, and then transfected into 293T cells using Lipo2000 reagent. After 36 hours, cells were collected, and samples were dosed according to the double luciferase assay (Promega). Then the two fluorescence signals were detected, and the relative fluorescence intensity was calculated.

### 2.10 Western blot

After culturing the transfected SK-RG cells for 48 hours, the total protein was extracted, the protein concentration was measured, and the loading amount was calculated. The total protein was separated by 6%-15% sodium dodecyl sulfate-polyacrylamide gel and transferred on a PVDF membrane, then blocked in 1×TBST solution of 5% nonfat dry milk for 2 h. Incubation was then performed with antibodies against mTOR (1:1000, Proteintech), p-S6K (1:1000, Proteintech), p-mTOR, P27(1:500; Bioworlde), cyclin D1 (1:500; Bioworlde), MMP9 (1:1000, Boster), MMP2 (1:1000, Bioss) and β-actin (1:1000, ZSGB-Bio). Added horseradish peroxidase-conjugated goat anti-rabbit antibody or goat anti-mouse antibody, and react at room temperature for 2 h. After developing with the luminescent agent, the gel imaging system took pictures, and the protein bands were analyzed in grayscale using Image J.

### 2.11 Statistical methods

Prism 8.0 statistical software was used for statistical analysis, unpaired-samples *t* test was used between the two samples, and the results of each group were repeated three times. *P*<0.05, indicating that the difference was statistically significant.

## 3. Results

### 3.1 The expression level of mTOR was increased in KSHV-infected SH-SY5Y cells (SK-RG)

**Figure 1:**
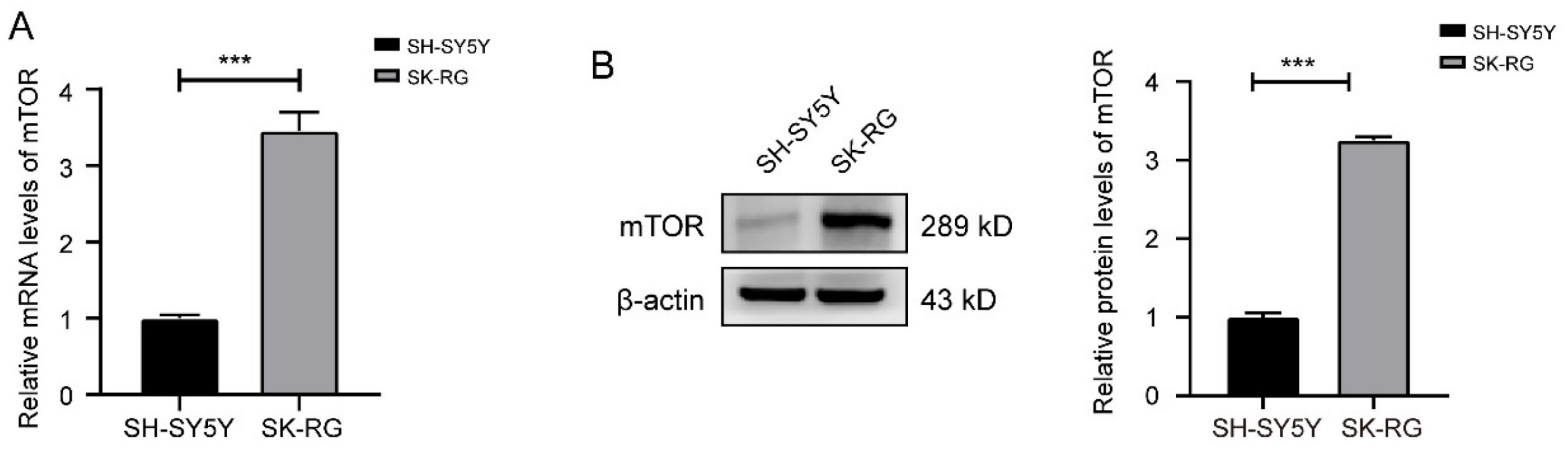
The mTOR levels was reduced in SK-RG cells. (A-B) The mTOR levels in SH-SY5Y and SK-RG cells were analyzed by real-time PCR and western blot. Data are presented as mean ± SD for three independent experiments. ***P < 0.001.

Based on the results of transcriptome sequencing performed from KSHV-infected and uninfected SH-SY5Y cells, we screened out significantly up-regulated and down-regulated genes. The expression of mTOR was also up-regulated in infected SK-RG cells, which was further verified by Western blot and real-time PCR assay (Figure 1A-B).

### 3.2 miR-769-3p can target and regulate the expression of mTOR in SK-RG cells

**Figure 2:**
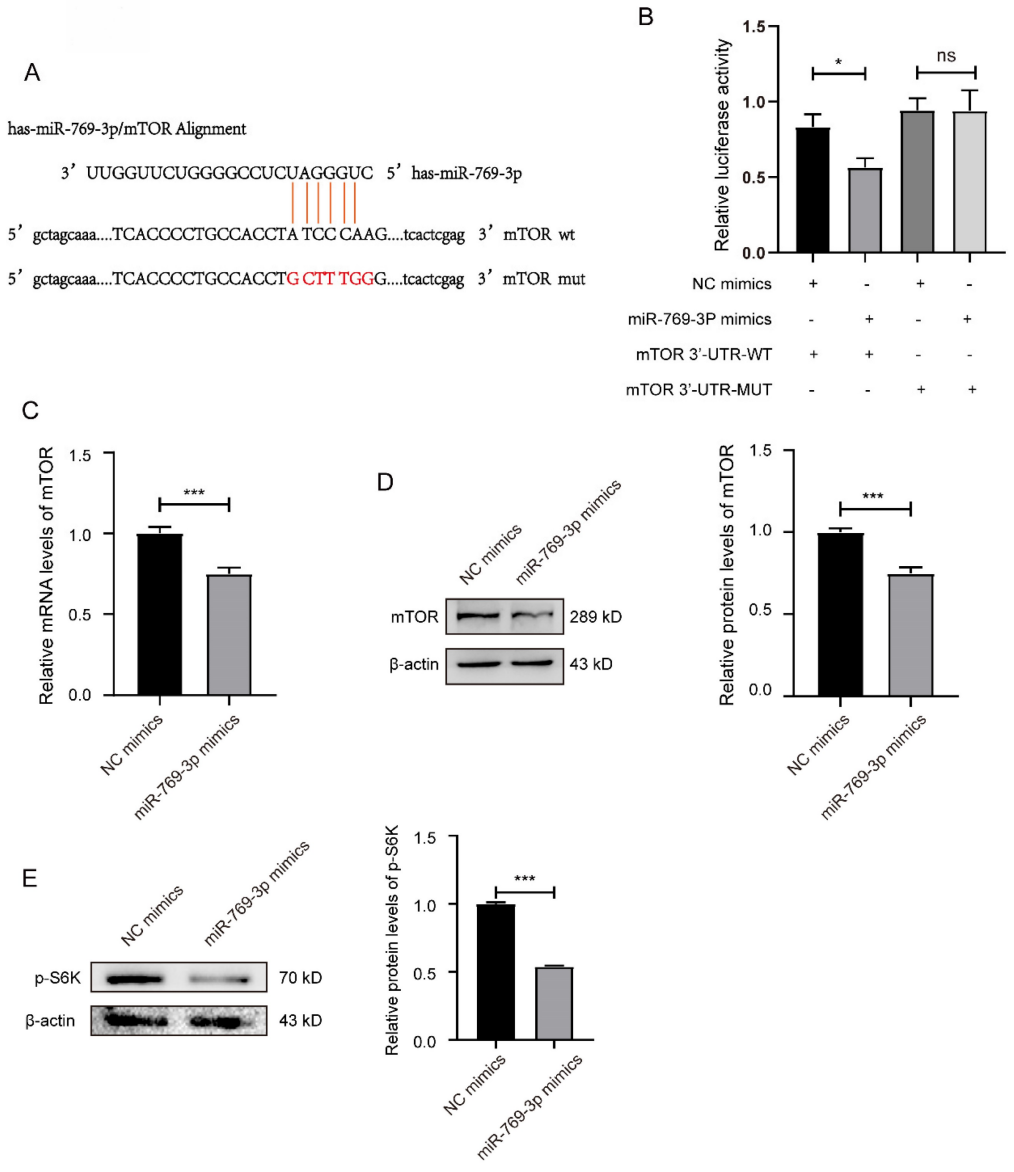
miR-769-3p had a targeting effect on the mTOR. (A) Potential binding sequences of miR-769-3p and the mTOR 3′-UTR were predicted using the Targetscan7.0 database. (B) The targeting relationship between miR-769-3p and mTOR was confirmed by the dual-luciferase reporter gene assay. (C) The mRNA expression level of mTOR was analyzed by the real-time PCR assay in miR-769-3p up-regulated SK-RG cells. (D) The protein expression level of mTOR was identified in miR-769-3p up-regulated SK-RG cells by Western blot. (E) The level of p-S6K protein in SK-RG cells after up-regulation of miR-769-3p was detected by western blot. Data are presented as Mean ± SD for three independent experiments. *P < 0.05; ***P < 0.001.

The bioinformatics were used to predict miRNAs that can target the mTOR. The results showed that the miR-769-3p could negatively regulate mTOR, and the potential binding sequences in the 3'-UTR region of mTOR mRNA were shown in the figure 2A. To confirm the binding of miR-769-3p to mTOR and its effect on proliferation and migration in SK-RG cells, the dual-luciferase reporter gene assay was performed. The results showed that co-transfection of miR-769-3p mimics and mTOR 3′-UTR-wt decreased the luciferase activity. However, the co-transfection of mTOR 3'-UTR-mut and miR-769-3p mimics did not change any luciferase activity (*P* < 0.05, Figure 2B). Western blot and real-time PCR results further demonstrated that the expression levels of mTOR decreased after up-regulated miR-769-3p in the cells. Moreover, the level of mTOR downstream target molecule p-S6K was also simultaneously decreased (Figure 2C-E). The above results indicate that miR-769-3p has a specific targeting effect on the expression regulation of mTOR.

### 3.3 Knockdown mTOR or overexpression of miR-769-3p inhibited the proliferation and cell cycle of SK-RG cells

**Figure 3:**
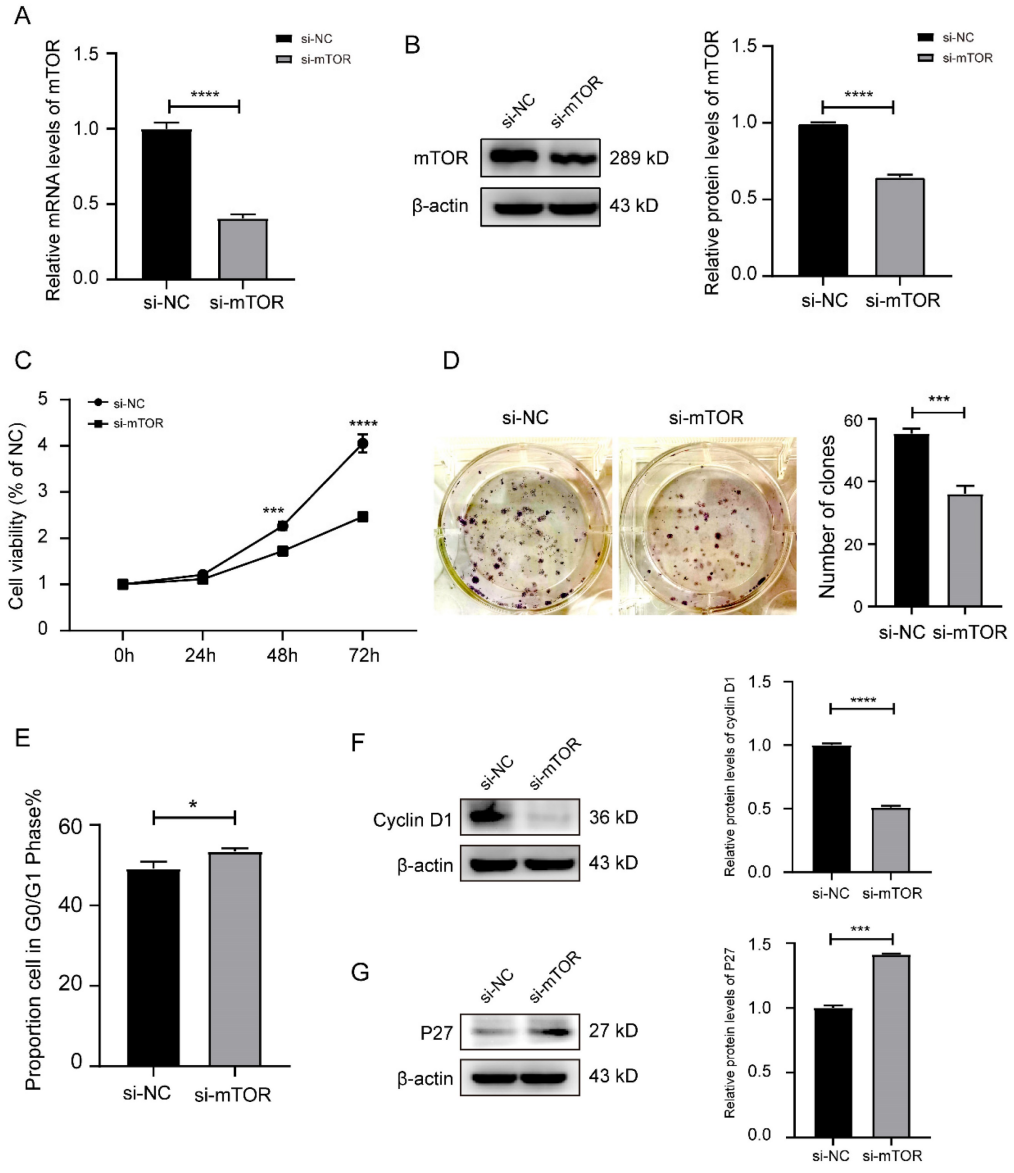
Knockdown of mTOR inhibited the cell proliferation and the cell cycle in SK-RG cells. (A-B) Detection of the mTOR interference efficiency by real-time PCR and Western blot. (C) The effect of mTOR knockdown on proliferation was detected by the MTT assay. (D) The effect of mTOR knockdown on the colony formation was detected by the plate clone assay. (E) The cell cycle was analyzed by the FCM in SK-RG cells after mTOR knockdown. The proportions of cells in the G0/G1, G2, and S phases were shown. (F and G) The protein expression levels of cyclin D1 and P27 were detected by western blot. Data are presented as mean ± SD for three independent experiments. *P < 0.05; ***P < 0.001; ****P < 0.0001.

We analyzed how knockdown of mTOR or overexpression of miR-769-3p would affect the proliferation and cell cycle in SK-RG cells. First, the transfection efficiency of si-mTOR was examined by real-time PCR and Western blot. The results showed that both mRNA and protein levels were significantly reduced after si-mTOR treatment (Figure 3A-B). The results of MTT and plate cloning assays showed that the knockdown of mTOR significantly inhibited the proliferation of SK-RG cells (Figure 3C-D). The cell cycle variation was studied using the flow cytometry and cycle-related proteins, and the results showed that the knockdown of mTOR significantly increased the proportion of cells in G0/G1 phase, resulting that SK-RG cell cycle arrest in G0/G1 phase (Figure 3E), the expression of cell cycle-related protein cyclin D1 was decreased while the expression of P27 was increased (Figure 3F-G).

**Figure 4:**
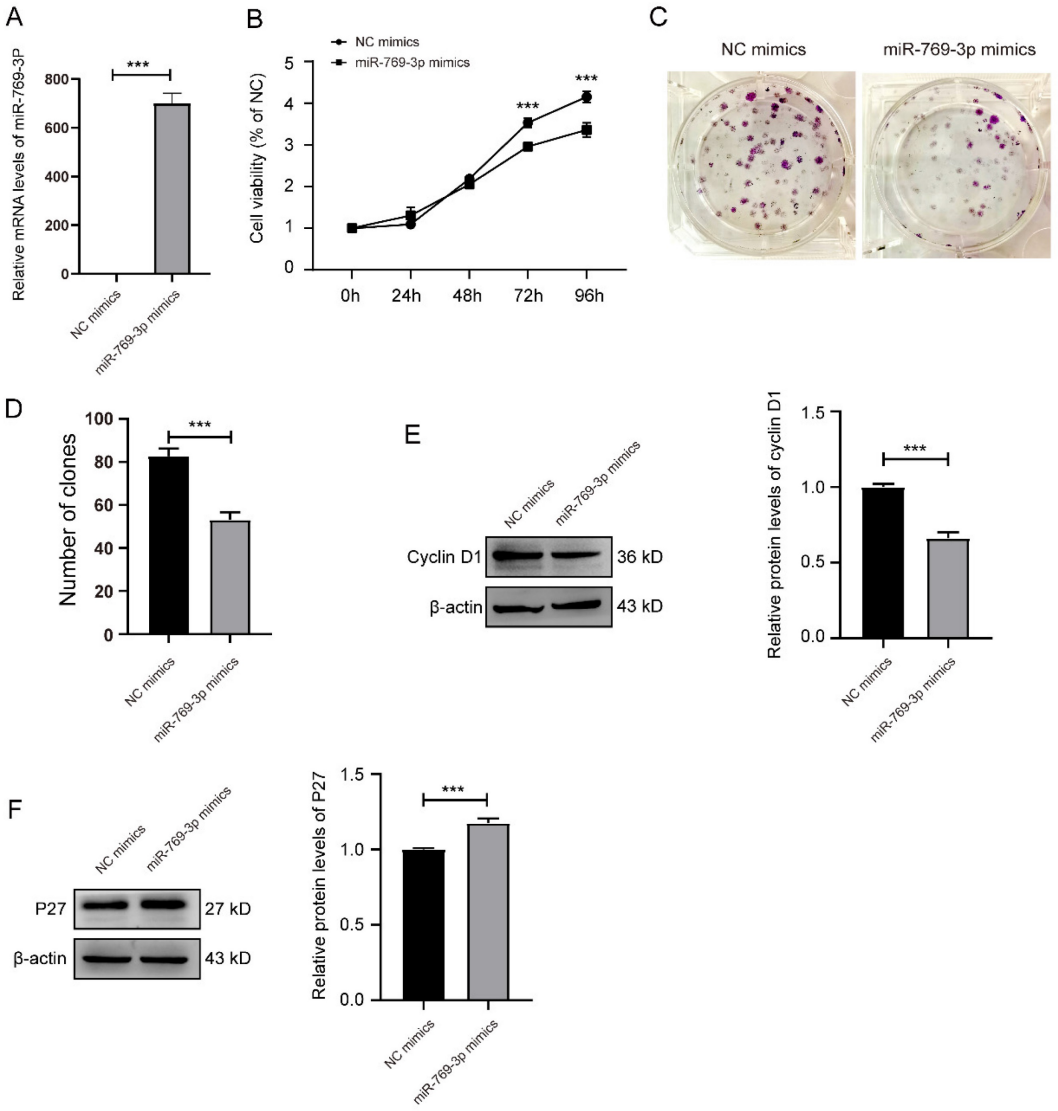
miR-769-3p inhibited the cell proliferation and the cell cycle in SK-RG cells. (A) Detection of the miR-769-3p up-regulated efficiency by real-time PCR. (B) The effect of miR-769-3p overexpression on proliferation was detected by the MTT assay. (C and D) The effect of miR-769-3p overexpression on the colony formation was detected by the plate clone assay. (E and F) The protein expression levels of cyclin D1 and P27 were detected by western blot. Data are presented as mean ± SD for three independent experiments. ***P < 0.001.

Subsequently, we examined the effect of miR-769-3p overexpression on SK-RG cells. Firstly, the transfection efficiency was determined by real-time PCR with a significantly increase after transfection (Figure 4A). Similar results to si-mTOR treatment on MTT and the plate cloning assays were obtained. The proliferation of SK-RG cells was inhibited after overexpression of miR-769-3p (Figure 4B-D), the expression of cyclin D1 was decreased and P27 was increased (Figure 4E-F).

### 3.4 Knockdown mTOR or overexpression of miR-769-3p inhibited the migration of SK-RG cells

**Figure 5:**
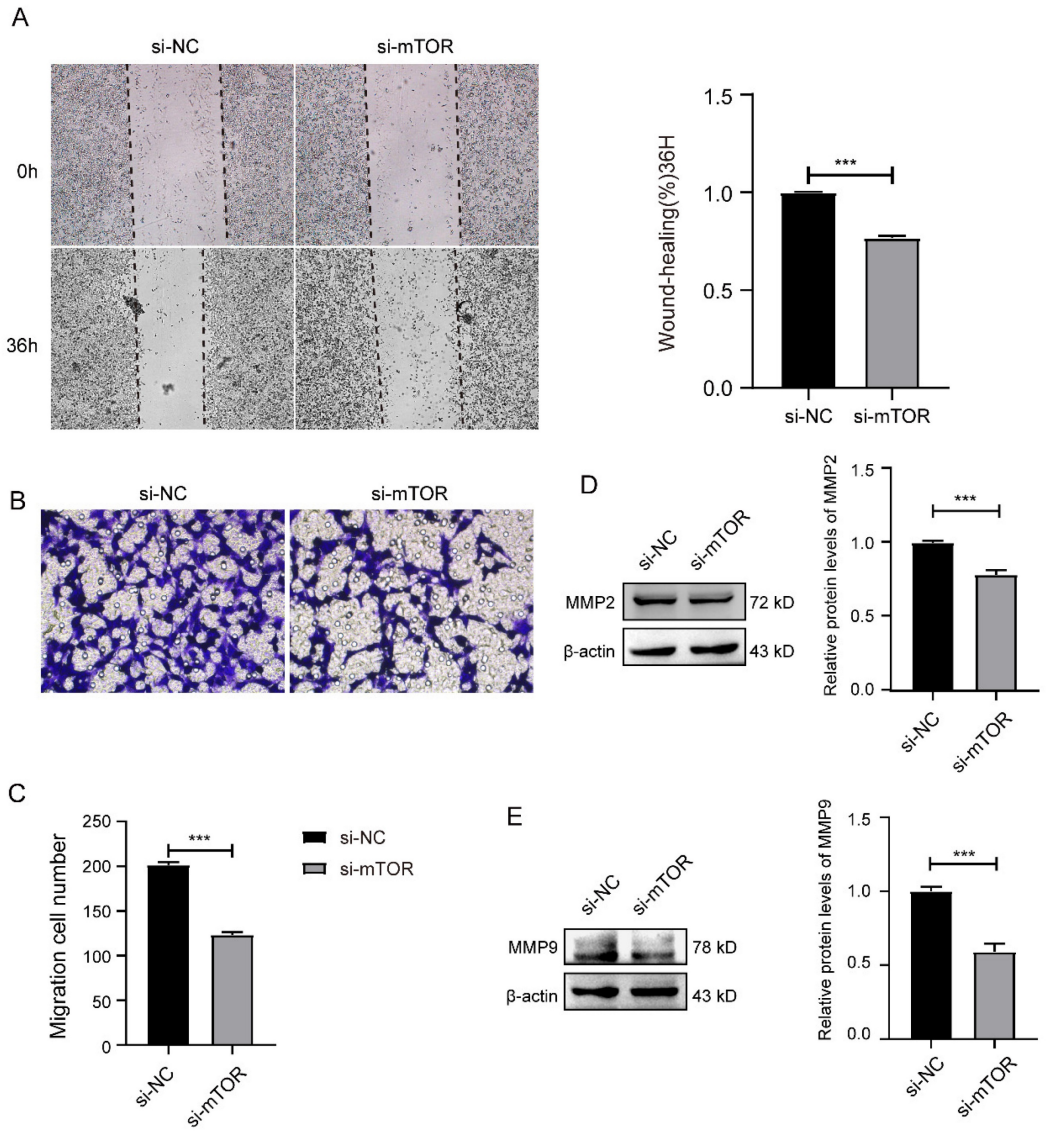
Knockdown of mTOR inhibited the cell migration in SK-RG cells. (A) The effect of mTOR knockdown on the migration was detected by the wound healing assay (40×). (B-C) The effect of mTOR knockdown on the migration was detected by the transwell assay (400×). (D-E) Protein levels of MMP2 and MMP9 were detected by western blot. Data are presented as Means ± SD for three independent experiments. ***P < 0.001.

Further, we analyzed whether knockdown mTOR could influence the migration of SK-RG cells. The results of the cell scratch assay showed that the relative gap width after knockdown of mTOR was larger than that in the control group (Figure 5A). In the transwell assay, the number of transmembrane cells after knockdown of mTOR was significantly less than that in the control group (Figure 5B-C). At the same time, in the western blot results, the expression of migration-related proteins MMP2 and MMP9 were lower than that in the control group after knocking down mTOR (Figure 5D-E).

**Figure 6:**
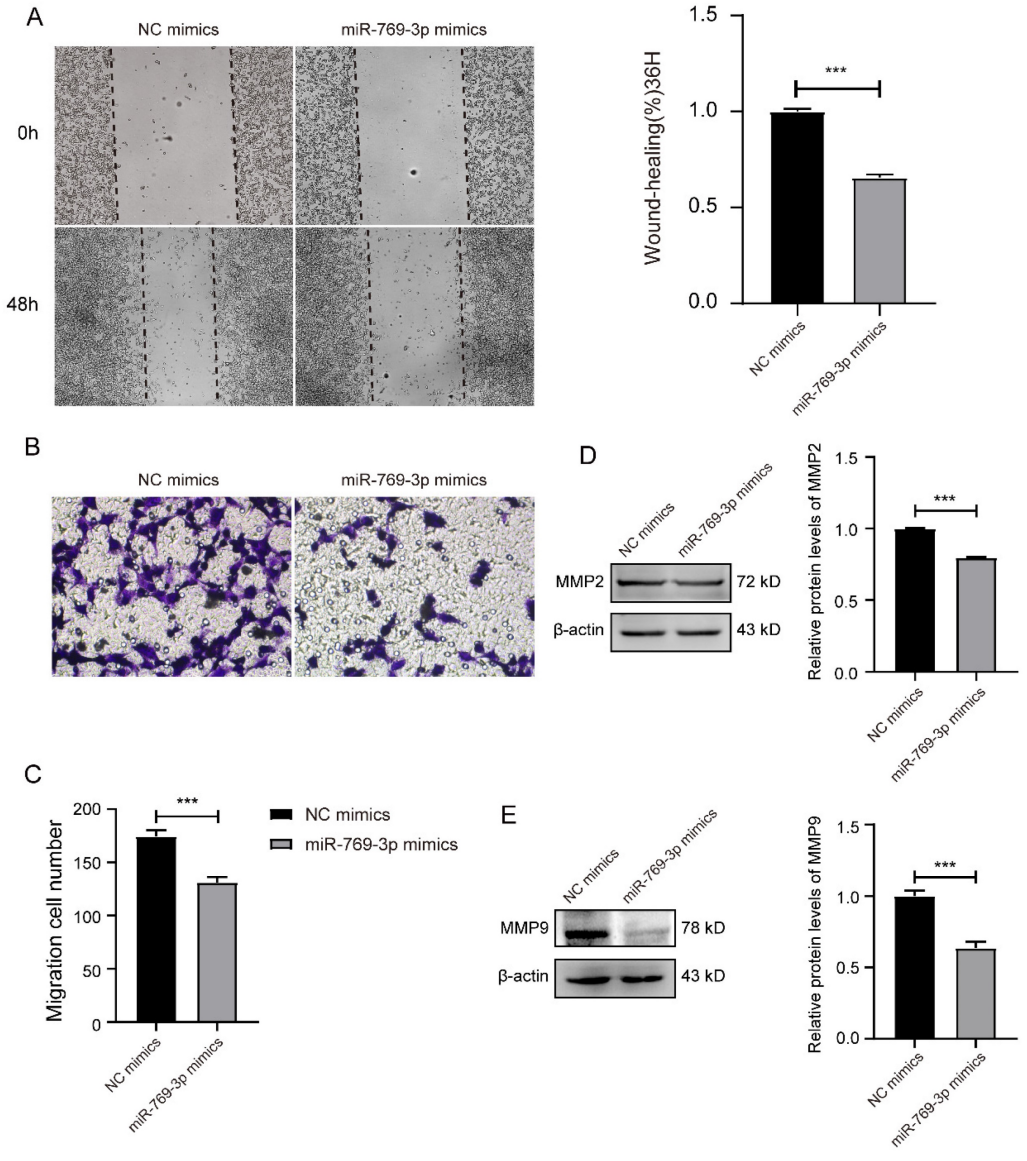
miR-769-3p inhibited the cell migration in SK-RG cells. (A) The effect of miR-769-3p overexpression on the migration was detected by the wound healing assay (40×). (B-C) The effect of miR-769-3p overexpression on the migration was detected by the transwell assay (400×). (D-E) Protein levels of MMP2 and MMP9 were detected by western blot. Data are presented as Means ± SD for three independent experiments. ***P < 0.001.

The similar results were observed in SK-RG cells after the overexpression of miR-769-3p. In this case, the relative gap width of scratches was larger than that of the control group (Figure 6A). The number of migrated cells in the transwell assay was less than that of the control group (Figure 6B-C) and the expressions of migration-related proteins MMP2 and MMP9 were also lower than those of the control group (Figure 6D-E). All the above data indicated that the knockdown of mTOR or overexpression of miR-769-3p could inhibit the migration ability of SK-RG cells.

### 3.5 Knockdown of mTOR or overexpression of miR-769-3p can reduce the expression of KSHV-related genes

**Figure 7:**
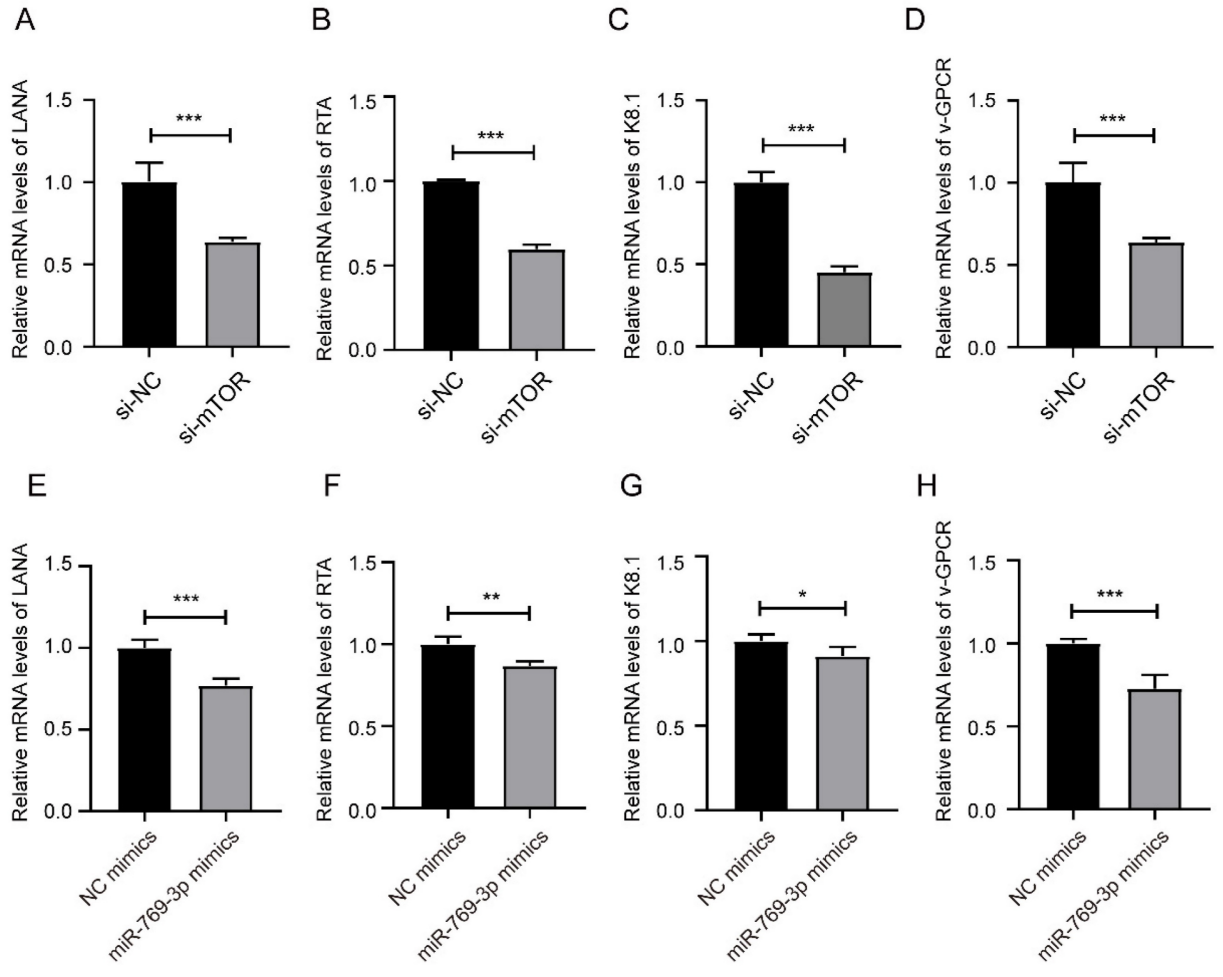
miR-769-3p and mTOR knockdown decreased the KSHV gene expression. (A-D) The mRNA expression levels of LANA, RTA, K8.1, and v-GPCR were analyzed by real-time PCR in the mTOR knockdown and control groups. (E-H) The mRNA expression levels of LANA, RTA, K8.1, and v-GPCR were analyzed by real-time PCR in the miR-769-3p overexpression and control groups. Data are presented as Mean ± SD for three independent experiments. *P < 0.05; **P < 0.01; ***P < 0.001.

After down-regulated mTOR or up-regulated miR-769-3p in SK-RG cells, we detected the mRNA levels of KSHV-related genes LANA, RTA, K8.1 and v-GPCR by real-time PCR. The results showed that the levels of LANA, RTA, K8.1 and v-GPCR genes were decreased after the knockdown of mTOR or overexpression of miR-769-3p (Figure 7A-H).

### 3.6 mTOR activator reversed the effects of miR-769-3p on SK-RG cell proliferation and migration

**Figure 8:**
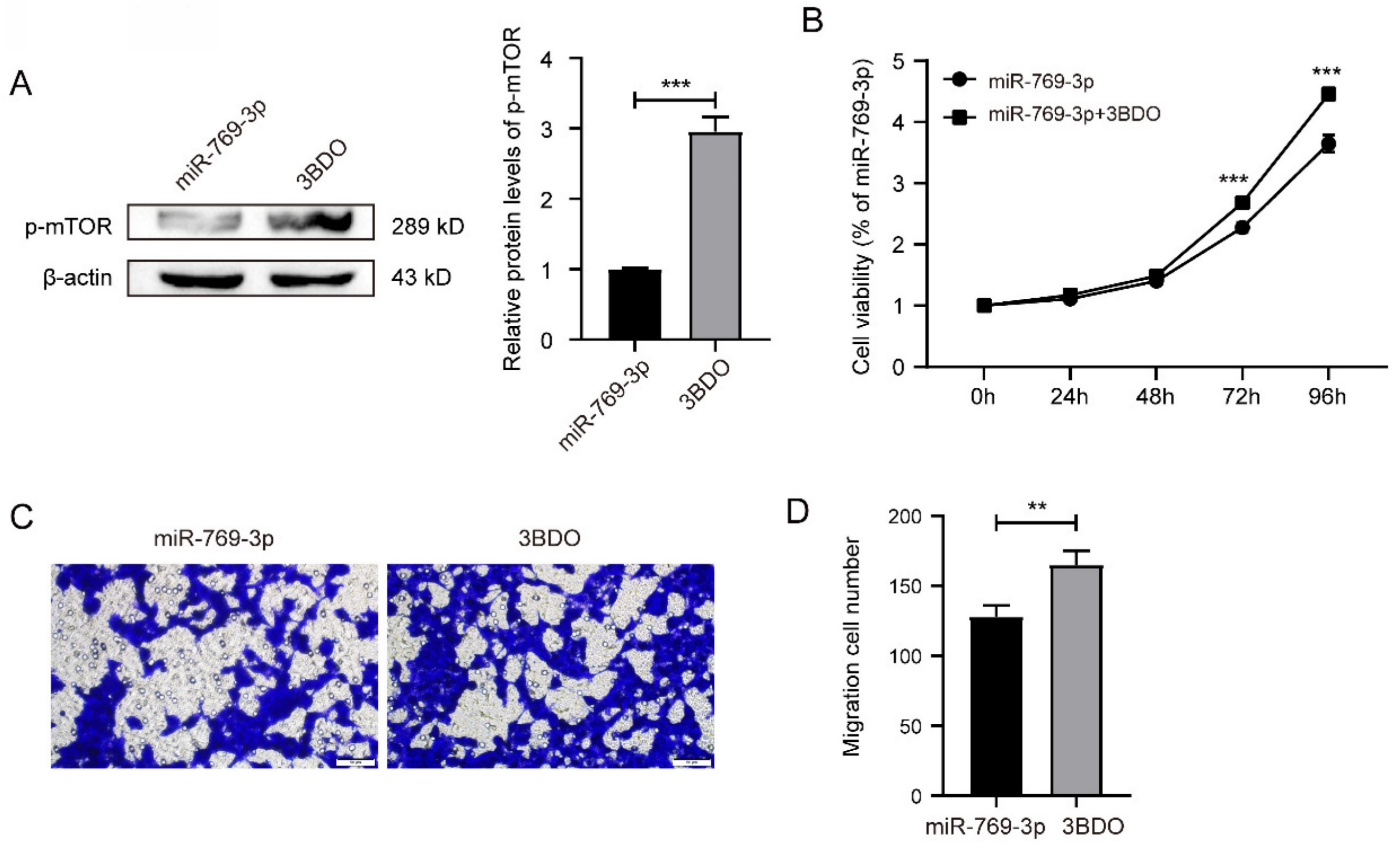
mTOR activator 3BDO reversed the inhibition of miR-769-3p on SK-RG cell proliferation and migration. (A) The Western blot analysis of p-mTOR protein in SK-RG cells treated with 3BDO. (B) The effect of activated mTOR on proliferation in the rescue assay was investigated by the MTT assay. (C-D) The effect of activated mTOR on the migration in the rescue assay was detected by the transwell assay (400×).

We used mTOR activator 3BDO to treat the miR-769-3p up-regulated SK-RG cells. The Western blot result revealed that the level of p-mTOR was up-regulated in 3BDO group, compared to the control (Figure 8A). Subsequently, MTT assays showed that 3BDO rescued and reduced the inhibitory effect of miR-769-3p on cell proliferation from 48 hours but more significantly after 72 hours (Figure 8B). Transwell assays results showed that the number of migrated cells after 3BDO treatment increased (Figure 8C-D). These results suggest that the mTOR activator has reversed the inhibitory effect of miR-769-3p on SK-RG cell proliferation and migration.

### 3.7 Comparison of the effects of miR-769-3p and Rapamycin on SK-RG cells

**Figure 9:**
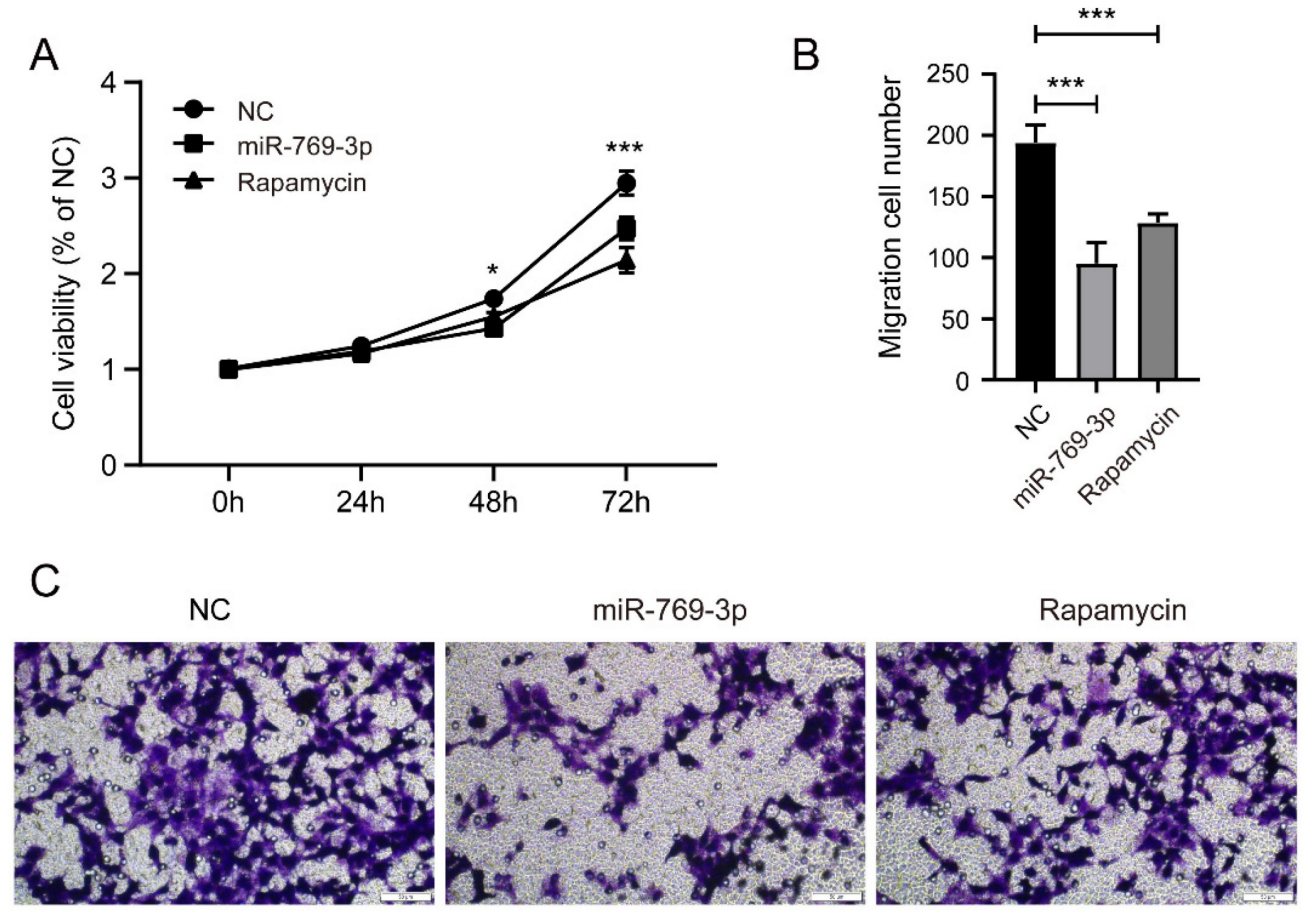
Comparison of the effects of miR-769-3p and Rapamycin on SK-RG cells. (A) The cell proliferation of miR-769-3p overexpressed or rapamycin treated SK-RG cells were detected by the MTT assay. (B-C) The cell migration of miR-769-3p overexpressed or rapamycin treated SK-RG cells were detected by the transwell assay.

To further determine the role of miR-769-3p on mTOR, we compared the proliferation and migration ability of SK-RG cells that were treated or untreated with rapamycin. The results of MTT and transwell assay showed that rapamycin had a similar inhibitory effect on SK-RG proliferation as miR-769-3p overexpression (figure 9 A), though its inhibitory effect on SK-RG migration was weaker than miR-769-3p (Figure 9 B-C).

## 4. Discussion

Like other herpesviruses, KSHV mainly exists in the pathological cells of KS, PEL and MCD in the latent form 30, 31. KSHV lytic activation usually occurs under the condition of immunosuppression. Many KSHV proteins related to the lytic state are expressed during this period, among which the expression of KSHV lytic switch protein transcriptional activator (RTA) is essential for this process 32-34. In our previous study, we have proved that KSHV could infect nerve cells and spontaneously activate the lytic state in these cells 5, 6, 10.

Many studies have reported that mTOR is abnormally activated in tumor cells, thereby promoting the growth, proliferation and metastasis of tumor cells 35-37. mTOR not only plays a key role in the cancer cell growth and metabolism, but also promotes their cell cycle by promoting the binding of the cyclin D1 to cyclin-dependent kinases (accelerating the process from G1 phase to S phase), thus facilitate the occurrence and development of tumors 38. While mTOR was found up-regulated in the KSHV infected nerve cell, the growth of the infected cells was also faster 6. Through various experimental observations on the interference and activation of mTOR in cells, we found that mTOR is indeed related to the proliferation of KSHV-infected nerve cells. After infection, KSHV can binds to receptors on target cells to activate the PI3K/AKT/mTOR pathway 39. It has been reported that in KSHV-infected endothelial cells, KSHV promotes viral replication by activating mTORC1^40^. Therefore, mTOR may be used as a target to inhibit the cleavage and replication of KSHV. In SK-RG cells, the proliferation ability of mTOR knocking down cells was inhibited, which was further verified by our rescue experiment.

In the endothelial cells of mice infected with the KSHV v-GPCR, mTOR knockdown can interfere with the paracrine transformation of v-GPCR by increasing the accumulation of dephosphorylated 4EBP, thereby inhibiting the tumor growth 41. During the lytic state, v-GPCR enhances the level of RTA by activating mTOR and enhancing the binding of SP1/SP3 and ORF50. At the same time, RTA can activate v-GPCR as a molecular switch during KSHV cleavage. This positive feedback pathway can promote the production of viral particles. These studies suggest that mTOR may be an important target in KSHV-infected cells. In this study, we found that mTOR was up-regulated in KSHV infected nerve cells. After knocking down mTOR, the ability of proliferation and migration were inhibited, and the expression of virus-related genes RTA, LANA, K8.1 and v-GPCR were reduced.

Through the predictive analysis of bioinformatics software, we predicted that miR-769-3p may have a regulatory effect on mTOR. Many studies have shown that miR-769-3p can inhibit the occurrence and development of tumors. For example, miR-769 can promote the proliferation of melanoma cells by targeting GSK3B in melanoma 42. miR-769-3p and SAHA synergistically promote GC cell apoptosis in gastric cancer 43. miR-769-3p can also inhibit the proliferation, migration and invasion of glioma cells 27. However, whether miR-769-3p had a regulatory effect on mTOR and the effect on KSHV-infected nerve cells was not reported before, which being of a value for further study. In this study, we confirm the regulatory effect of miR-769-3p on mTOR using the dual fluorescein reporter gene assay followed by overexpression assay of miR-769-3p in SK-RG cells, and proved that the cell proliferation and migration abilities were inhibited, and the expression of virus-related gene RTA, LANA, K8.1 and v-GPCR were also reduced. 3-benzyl-5-((2-nitrophenoxy) methyl)-dihydrofuran-2(3H)-one (3BDO), was a chemical small molecule that activated mTOR to inhibit autophagy in human umbilical vein endothelial cells (HUVECs) and neuronal cells 44. 3BDO can active the PI3K/AKT/mTOR pathway to enhance chondrocyte-specific gene degradation to protect chondrocytes 45. Thus, we used 3BDO in SK-RG cells upregulated by miR-769-3p to further verify the targeting relationship between miR-769-3p and mTOR, and found that mTOR and p-mTOR protein expression level and cell proliferation, migration ability were upregulated, which indicated that mTOR reversed the inhibitory effect of miR-769-3p on the proliferation and migration of SK-RG cells.

Finally, after treating with the mTOR inhibitor, rapamycin, we found that miR-769-3p and rapamycin had similar inhibitory effects on SK-RG cell proliferation, but in terms of migration, miR-769-3p was slightly stronger than rapamycin. This indicates that the inhibition of miR-769-3p on SK-RG cell migration not only depends on mTOR, but also may be related to other molecules, which need to be further explored. There is no doubt that the miRNA as small molecule drugs have lower cytotoxicity, which suggests that miR-769-3p has potential to be developed as relevant therapeutic drugs.

In summary, we proved that miR-769-3p inhibits the proliferation and migration of KSHV-infected neuronal cells by targeting mTOR, and inhibited the expression of KSHV virus genes. Our findings suggested that miR-769-3p plays a key role in controlling the malignant development of KSHV-infected neuronal cells and that it is possible to consider increasing the level of this small molecule nucleic acid, and select carrier delivery to make nanomolecular drugs for therapy of KSHV infection in clinic.

## Figures and Tables

**Table 1:** Primer sequences

Primer name	Primer sequence
LANA-F Primer	5′-AGCCACCGGTAAAGTAGGAC-3′
LANA-R Primer	3′-GATGTGACCTTGGCGATGAC-5′
RTA-F Primer	5′-GAGTCCGGCACACTGTACC-3′
RTA-R Primer	3′-AAACTGCCTGGGAAGTTAACG-5′
K8.1-F Primer	5′-AAAGCGTCCAGGCCACCACAGA-3′
K8.1-R Primer	3′-GGCAGAAAATGGCACACGGTTAC-5′
v-GPCR-F Primer	5′- GTGCCTTACACGTGGAACGTT-3′
v-GPCR-R Primer	3′- GGTGACCAATCCATTTCCAAGA -5′
mTOR-F Primer	5′-GCTAGGTGCATTGACATACAACA-3′
mTOR-R Primer	3′-AGTGCTAGTTCACAGATAATGGC-5′
β-actin-F Primer	5′-CGGAACCGCTCATTGCC-3′
β-actin-R Primer	3′-ACCCACATCGTGCCCATCTA-5′
miR-769-3p-RT primer	5′GTCGTATCCAGTGCAGGGTCCGAGGT ATTCGCACTGGATACGACAACCAA -3′
miR-769-3p-F primer	5′-GCTGGGATCTCCGGGGTC-3′
miR-769-3p-R primer	5′-AGTGCAGGGTCCGAGGTATT-3′
U6-RT primer	5′GTCGTATCCAGTGCAGGGTCCGAGGTATT CGCACTAGATACGACAAAATA-3′
U6-F primer	5′-AGAGAAAGATTAGCATGGCCCCTG-3′
U6-R primer	5′- ATCCAGTGCAGGGTCCGAGG -3′
